# Patellofemoral joint forces during running are not different between adolescents with and without patellofemoral pain—a cross-sectional study

**DOI:** 10.3389/fspor.2026.1762881

**Published:** 2026-03-05

**Authors:** Natalie Mazzella, Danielle Trowell, Aaron Fox, Natalie Saunders, Bill Vicenzino, Jason Bonacci

**Affiliations:** 1 Centre for Sport Research, Institute for Physical Activity and Nutrition, Deakin University, Geelong, VIC, Australia; 2 La Trobe Sport and Exercise Medicine Research Centre, La Trobe University, Bundoora, VIC, Australia; 3 Centre for Sport Research, Institute for Physical Activity and Nutrition, Deakin University, Burwood, VIC, Australia; 4 School of Health and Rehabilitation Sciences, The University of Queensland, St Lucia, QLD, Australia

**Keywords:** biomechanics, gait, musculoskeletal, patellofemoral pain, youth

## Abstract

**Introduction:**

The purpose of this study was to determine if adolescents (aged 12–18 years) with patellofemoral pain demonstrate greater peak and cumulative patellofemoral joint forces when compared to asymptomatic adolescents during running.

**Methods:**

Twenty-six adolescents with patellofemoral pain (14 male, 12 female, mean *±* standard deviation age: 14.4 *±* 1.7 years) and 24 asymptomatic adolescents (13 male, 11 female, mean ± standard deviation age: 14.1 *±* 1.6 years) participated in this cross-sectional study. Participants ran on an instrumented treadmill in a traditional athletic shoe while kinematic and kinetic data were collected. Peak knee flexion angle, peak internal knee extension moment, and cumulative and peak patellofemoral joint force were compared between groups using a one-way analysis of covariance (*α* = 0.05). The mean difference (MD) with 95% confidence intervals [95% CI] and standardised mean differences (SMD) were calculated to express the magnitude of difference between groups.

**Results:**

Peak patellofemoral joint force [MD = −0.22 (−0.33, 0.77) N/kg] and cumulative patellofemoral joint force [MD = −6.26 (−37.47, 50.00) Bw.s/km] were not different between adolescents with patellofemoral pain and asymptomatic adolescents. Peak knee flexion [MD = 0.19 (−2.72, 2.33) deg] and knee extension moment [MD = −0.11 (−0.07, 0.29) Nm/kg] were also not different between groups.

**Discussion:**

Greater patellofemoral joint forces during running are not evident in adolescents with patellofemoral pain when compared to asymptomatic adolescents.

## 1 Introduction

Patellofemoral pain (PFP) is a prevalent cause of knee pain in adolescents (1). Previously considered to be self-limiting, long-term studies purport that PFP developed during adolescence is persistent and associated with poor long term health outcomes (2, 3). Adolescents with PFP participate in less physical activity, experience poorer mental health, and report lower quality of life when compared to pain-free adolescents (2, 4, 5). Cessation of physical activity is evident in more than 70% of adolescents with PFP (2). Pain is often longstanding in this age group with longitudinal cohort studies estimating 19% continue to experience pain six years on from their initial diagnosis (3). While numerous studies have endeavoured to identify a framework that may explain the development of PFP, these studies have typically focused on features identified in adults with PFP (6, 7). In order to improve the long-term trajectory of adolescent PFP, identification of contributing factors is necessary.

The aetiology of PFP is complex and multifactorial (7, 8). Elevated patellofemoral joint (PFJ) forces have been proposed as a contributing factor to the development of PFP (7, 8). Specifically, higher forces may lead to microstructural tissue damage within the PFJ and surrounding tissues (9). Greater PFJ reaction forces have been shown to elevate joint tissue stress (10, 11). Signs of elevated PFJ stress have been observed in adults with PFP including increased water content within the PFJ (12), and bone marrow oedema on magnetic resonance imaging (13, 14). Repetitive submaximal trauma may contribute to microscopic joint changes which exceed the joint tissue capacity to tolerate load, leading to the development of symptoms (15). As such, the reduction of joint force has been advocated in the management of PFP (16, 17).

A strategy to address PFJ force has been the manipulation of lower limb biomechanics. Lower limb biomechanical changes can alter contact forces and force distribution within the PFJ (7, 18). Adults with PFP have demonstrated alterations in lower limb kinematics, such as an increase in hip adduction and greater contralateral pelvic drop (19, 20), that can elevate PFJ force. Yet, limited research has investigated whether the same biomechanical differences exist in adolescents. An association between dynamic knee valgus and PFP has been observed in children and adolescents during a lateral step-down task (21). However, to date, only one study has compared the biomechanics of adolescents with and without PFP during walking and running (22). Adolescents with PFP ran with a higher cadence and greater knee flexion than asymptomatic adolescents, but PFJ forces were not measured. Cadence and knee flexion have been shown to influence PFJ forces in adults with and without PFP during running (23, 24). Greater knee flexion during stance increases the demand on the quadriceps leading to a higher knee extension moment and PFJ force (23). It is unclear if adolescents with PFP exhibit different PFJ forces to their asymptomatic counterparts. Cumulative joint forces from repetitive lower limb activities may contribute to the development or persistence of PFP in adolescents. Cumulative joint force is a by-product of the magnitude of joint force, as well as the number of loading cycles applied to the joint (25–27). Considering the poor long-term health outcomes associated with adolescent PFP, the implementation of effective treatment strategies early is imperative. The aim of this study was to determine if adolescents with PFP demonstrate differences in peak and cumulative PFJ forces when compared to asymptomatic adolescents during running. A secondary aim was to identify if adolescents with PFP demonstrated differences in knee joint kinematics and kinetics when compared to asymptomatic adolescents during running. It was hypothesized that adolescents with PFP would demonstrate higher PFJ loads when compared to asymptomatic adolescents during running.

## 2 Materials and methods

Adolescents with PFP and asymptomatic adolescents participated in this study. This study was conducted according to the STROBE (Strengthening the reporting of observational studies in epidemiology statement—guidelines for reporting observational studies) guidelines for observational studies (28). Ethical approval was granted by the Deakin University human ethics committee (2021-135). Written informed consent was obtained from all participants, and their parent or guardian for those under the age of 18, prior to the collection of data. Since no studies have investigated PFJ reaction force in adolescents with PFP, a previous study reporting peak PFJ reaction force in adults was used for an a-priori sample size calculation (29). To determine a difference between groups at an *α* of 0.05, *β* = 0.8 and with an effect size of 0.8, we required at least 21 participants per group (G*Power 3.1.9.7, Heinrich-Heine University, Germany).

Adolescents with PFP were recruited to participate through advertisements placed across social media and local sports medicine clinics and footwear stores from January 2022 to September 2022. Adolescents with PFP were physically screened to confirm the diagnosis of PFP by an experienced healthcare practitioner (NM). Adolescents with PFP were included if they: (i) were aged 12–18 years, (ii) had retropatellar knee pain from a non-traumatic onset of at least 6 weeks duration, (iii) experienced pain exceeding 3/10 on a numeric pain rating scale during the week prior, (iv) had pain which was provoked by at least two activities that load the PFJ (e.g., squatting, stair ascent or descent, prolonged sitting, running), and (v) had pain on palpation of the patellar facet or during a double leg squat. Exclusion criteria were: (i) a history of lower limb, hip, or spine surgery, (ii) concomitant pain or pathology of other knee structures (e.g., menisci, patella tendon, iliotibial band, tibial tuberosity), (iii) pain or injury in the foot, ankle, hip, pelvis, or lumbar spine, and (iv) diagnosis of other forms of knee pathology (e.g., Sinding Larsen syndrome, Osgood Schlatter's Disease). Asymptomatic adolescents were included if they were: (i) aged between 12–18 years, (ii) had no history of knee, foot, hip, or lower limb pain, and (iii) had no history of lower limb surgery, hip, or spine surgery. Participant groups were matched according to sex, body mass index and participation in physical activity.

Adolescents attended the Deakin University 3D Gait laboratory for one session of data collection. Participants were provided a five-minute period of familiarisation and self-selected warm up on the treadmill. Participants were required to run in a traditional athletic shoe for four minutes on an instrumented treadmill at a controlled speed between 2.0 and 3.0 m/s which was consistent with participant's self-selected speed during the warm-up. The traditional athletic shoe was the Asics Cumulus (Asics, Kobe, Japan). The Asics Cumulus is a traditional athletic shoe with a 10 mm heel toe offset and a mass of 285 g (Asics, Kobe, Japan).

For participants with PFP, usual and worst pain severity over the past week was obtained using an 11-point numeric rating scale (30), and self-reported symptom and functional limitations obtained with the anterior knee pain scale (31), and the knee injury and osteoarthritis outcome score—patellofemoral (KOOS-PF) subscale (32). To identify participants stage of pubertal development, participants completed the modified pubertal maturational observational scale (33, 34). This scale can reliably classify adolescent developmental stages in a non-invasive manner and has been used in other studies of adolescent running biomechanics (35, 36).

**Figure 1:**
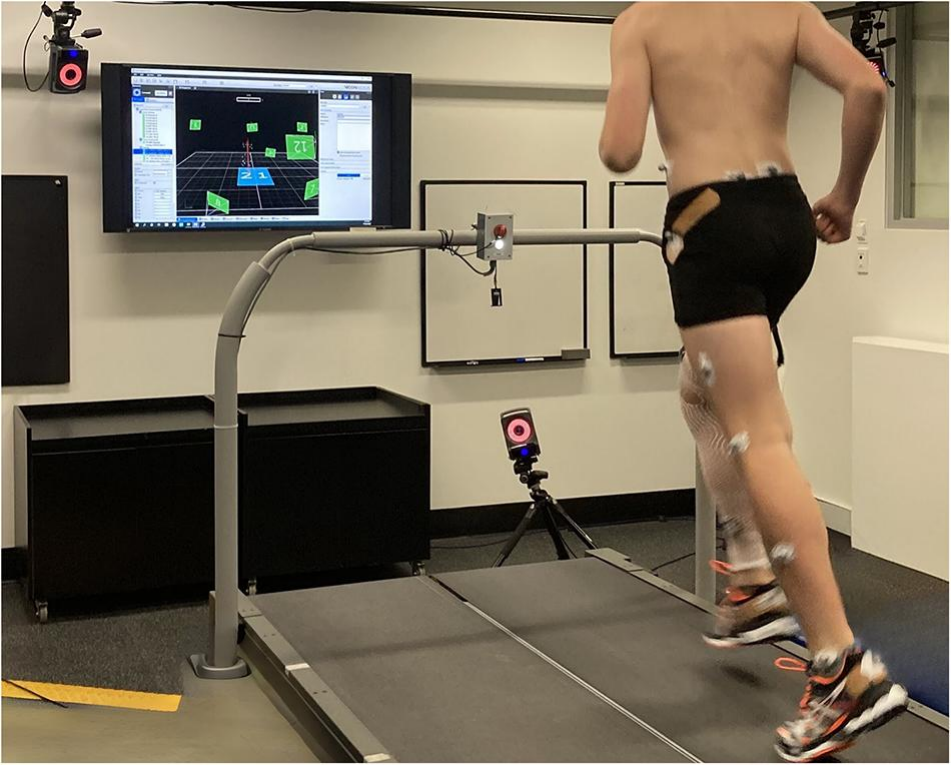
Representative participant running on the instrumented treadmill during three-dimensional motion capture analysis.

Three-dimensional kinematics of the lower limb were measured using an eight camera VICON motion analysis system (Oxford Metrics, Oxford, England) sampling at 250 Hz collected in synchrony with ground reaction force (GRF) data from an instrumented treadmill (Bertec, Ohio, USA) sampling at 1,000 Hz (see Figure 1). Prior to the collection of running trials, a static calibration trial was collected with participants standing in a neutral anatomical position. Thirty-two 14 mm reflective markers were taped to participants on the following anatomical landmarks in accordance with an established lower limb model (37). Markers were placed bilaterally on the iliac crest, anterior and posterior superior iliac spines, greater trochanter, anterior and lateral thigh, medial and lateral epicondyles of the femur, proximal, lateral, and distal tibia, medial and lateral malleolus, calcaneus, and the base of the third and fifth metatarsals. Markers on the medial epicondyle of the femur and the medial malleolus were removed for running trials.

Marker trajectory and GRF data were reconstructed in VICON Nexus software Version 2.12.1 (Oxford Metrics, Oxford, England), before processing using custom MATLAB (version 2021a; The Mathworks Inc, Natick, MA) scripts and OpenSim 4.3 software (OpenSim, Stanford, California, USA). Experimental marker trajectories and GRF data were low-pass filtered using a zero-lag fourth-order Butterworth filter with a cutoff frequency of 20 Hz (38, 39). OpenSim software was used to create participant-specific musculoskeletal (MSK) models by scaling a generic MSK model to match the participants' static calibration trials based on experimental marker positions (40). MSK models were combined with marker data from dynamic trials to calculate joint kinematics and kinetics using the inverse kinematics and dynamics tools, respectively. A 60 N vertical GRF threshold was used to identify gait events (41). For participants with PFP, data were analysed from their symptomatic limb as per previous studies performed in those with PFP (19, 42). For any participants experiencing bilateral symptoms, data were analysed from the most painful limb. Data were analysed from the dominant limb for asymptomatic adolescents which is consistent with previous biomechanical studies of running (22, 43).

To calculate PFJ forces, a previously described biomechanical model was used (44, 45). PFJ reaction force was estimated by multiplying the estimated quadriceps force by a constant that defines the relationship between PFJ reaction force and knee flexion angle (46). To calculate quadriceps force for each knee flexion angle, the net knee extensor moment was divided by the effective lever arm for the quadriceps. To determine the quadriceps effective lever arm, a non-linear equation was fit to the data of van Eijden et al. (46), and the output of the model was PFJ reaction force. This approach has commonly been used to estimate PFJ reaction force in previous biomechanical studies and is sensitive to detect changes in PFJ reaction force between people with and without PFP (47, 48). This model was deemed appropriate to use in an adolescent cohort as there is no direct evidence that input parameters for the model differ between adults and adolescents.

Stride length was calculated as the anterior-posterior distance between successive heel marker positions at initial contact, plus the distance that the treadmill belt moved between each foot contact (22). Cadence was calculated by determining the number of foot strikes per minute. PFJ impulse was calculated per stride as the integral of PFJ reaction force over time. Cumulative PFJ force was estimated as the total force per one kilometre of continuous running. This was calculated by multiplying the impulse per stance (sum of positive and negative) by the number of steps taken to complete one kilometre of running (25, 49). Data were time-normalised to 0%–100% of the gait cycle and averaged over 20 strides from the final minute of the run. Outcome variables of interest included: (i) peak PFJ force, (ii) cumulative PFJ force, (iii) PFJ impulse, (iv) peak knee flexion angle, (v) peak knee extension moment, (vi) cadence, and (vii) stride length.

Participant age, height, body mass, body mass index, and running speed were compared between groups using independent *t*-tests. To assess differences in outcome measures between groups one-way analysis of covariance tests were performed in SPSS Version 29.0 (IBM, Chicago, USA), with group as the fixed factor and running velocity as the covariate. Assumptions of normality were examined using Shapiro Wilk and Levene's test. The mean difference (MD), 95% confidence intervals (CI) and standardised mean difference (SMD) were calculated for all outcome measures of interest. The SMD was calculated to express the magnitude of difference between groups and interpreted according to the following criteria: SMD 0.2–0.5: small change; SMD ≥ 0.5: moderate change; SMD ≥ 0.8: large change (50).

## 3 Results

**Figure 2:**
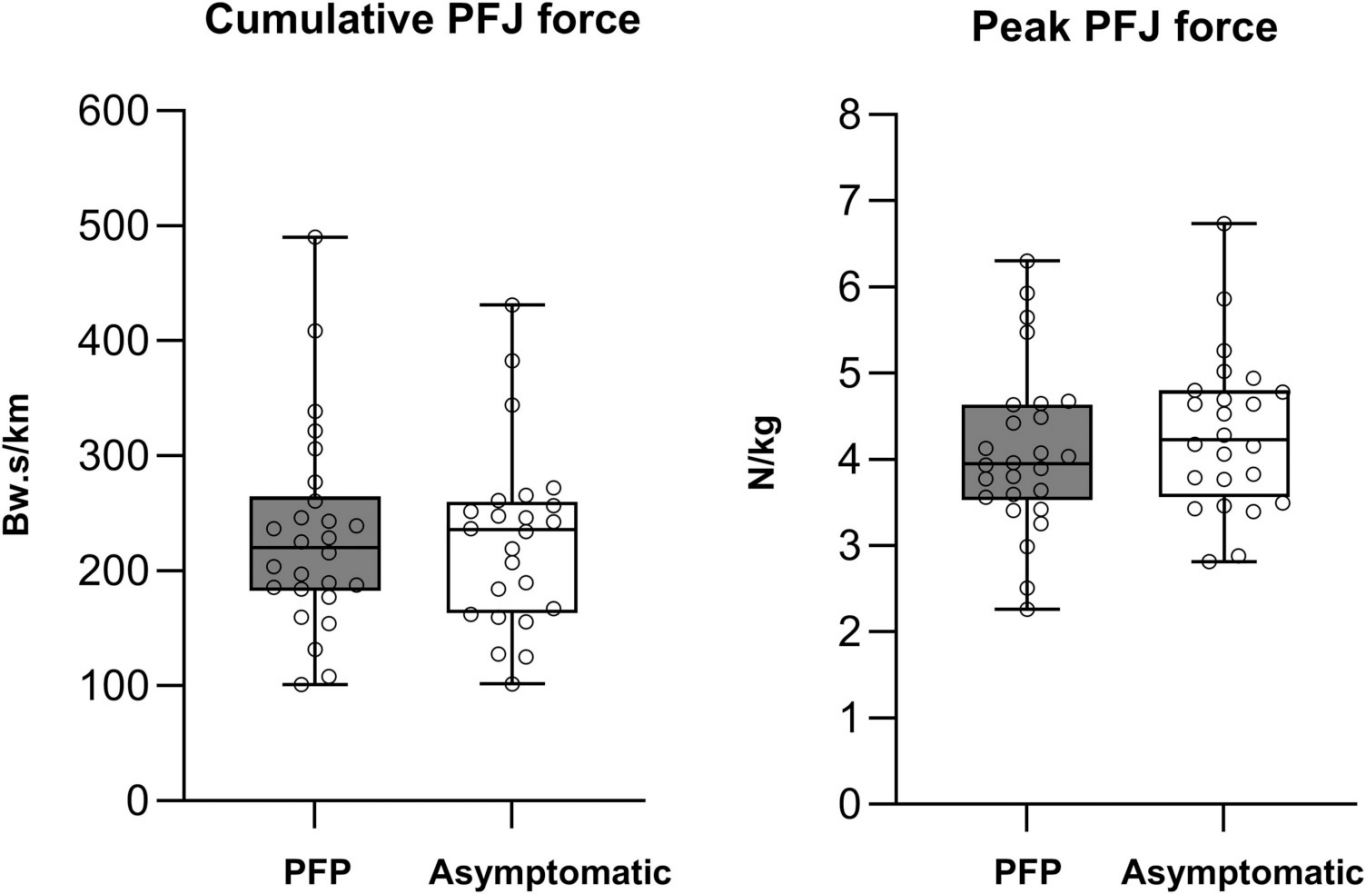
Peak and cumulative patellofemoral joint force between groups. BW, body weight; Kg, kilograms; N, newtons; PFJ, patellofemoral joint; s, seconds.

Twenty-six adolescents with PFP and 24 asymptomatic adolescents participated in this study. Participant demographics, physical activity and function are presented in Table 1. All participants had running experience and participated in a range of running related court and field-based sports. There were no differences in age (*p* = 0.47), height (*p* = 0.28), body mass (*p* = 0.94), body mass index (0.31) and physical activity (*p* = 0.13) between groups (see Table 1). Twenty-three adolescents with PFP and 24 asymptomatic adolescents had commenced or completed puberty. Mean running velocity was not different between the PFP cohort (2.38 *±* 0.19 m/s) and the asymptomatic cohort (2.43 *±* 0.24 m/s) (*p* = 0.36). During running, there was no difference in peak knee flexion, the internal knee extension moment or PFJ impulse between groups (Table 2). Similarly, peak and cumulative PFJ reaction force was not different between groups [MD = −0.22 [−0.33, 0.77] N/kg, SMD = 0.23, *p* = 0.42; and MD = −6.26 [−37.47, 50.00] Bw.s/km, SMD = 0.04, *p* = 0.78 respectively, Figure 2]. Adolescents with PFP ran with a higher cadence (SMD = 0.81, *p* = 0.01) and a shorter stride length (SMD = 0.57, *p* = 0.01) when compared to asymptomatic adolescents.

**Table 1:** Participant characteristics. Values are described as mean *±* SD unless otherwise stated.

Characteristics	PFP, *n* = 26	Asymptomatic, *n* = 24
Age, years	14.4 *±* 1.7	14.1 *±* 1.6
Sex, male (%)	14 (53.8)	13 (54.2)
Height, cm	167.8 *±* 10.0	170.9 *±* 9.7
Body mass, kg	64.1 *±* 13.3	63.9 *±* 13.6
Body mass index, kg/m^2^	22.6 *±* 3.4	21.7 *±* 3.1
Participation in physical activity, yes (%)	23 (88.5)	23 (95.8)
Physical activity participation, times/week	4.0 *±* 2.1	4.8 *±* 1.6
Duration of symptoms, months	24.8 *±* 20.4	N/A
Number with bilateral symptoms, *n* (%)	20 (76.9)	N/A
Worst pain^a^	6.7 *±* 1.6	N/A
Usual pain^a^	3.6 *±* 1.5	N/A
Anterior knee pain scale^b^	70.2 *±* 11.0	N/A
KOOS-PF^c^	52.4 *±* 13.8	N/A

^a^ Worst & Usual pain recorded on an 11-point numerical rating scale, 0 = no pain, 10 = worst pain imaginable.

^b^ Anterior knee pain scale, ranges from 0 to 100 points with higher scores indicating less knee disability.

^c^ Knee Injury and Osteoarthritis Outcome Score—Patellofemoral subscale, ranges from 0 to 100 points with higher scores indicating less knee disability.

**Table 2:** Spatiotemporal, patellofemoral and knee joint kinematics and kinetics across groups. Values are described as mean ***±*** SD unless otherwise stated.

Variable	PFP, *n* = 26	Asymptomatic, *n* = 24	MD [95% CI]	SMD	*p*
Stride length (m)	1.74 *±* 0.16	1.84 *±* 0.18	−0.06 [−0.01, −0.1]	0.57	0.01
Cadence (steps/min)	168.00 *±* 6.73	162.57 *±* 6.58	5.65 [1.81, 9.48]	0.81	0.01
Peak knee flexion angle (deg)	47.46 *±* 4.94	46.95 *±* 4.02	0.19 [−2.72, 2.33]	0.11	0.88
Peak knee extension moment (Nm/kg)	1.77 *±* 0.31	1.89 *±* 0.32	−0.11 [−0.07, 0.29]	0.38	0.22
PFJ impulse (N·s)	0.40 *±* 0.14	0.42 *±* 0.14	−0.03 [−0.06, 0.11]	0.12	0.54

CI, confidence interval; MD, mean difference; PFJ, patellofemoral joint, PFP, patellofemoral pain; SMD, standardised mean difference.

## 4 Discussion

Contrary to our hypothesis, adolescents with PFP did not demonstrate differences in peak knee joint kinematics, kinetics, or peak and cumulative PFJ force when compared to asymptomatic adolescents.

Our findings are similar to those observed during running in a cohort of adults with and without PFP (51). In that study, adults with PFP did not have greater peak PFJ force during running when compared to asymptomatic adults (51). Elevated PFJ force is thought to increase PFJ stress (52), and is therefore often a focus of PFP management (17). In contrast to our study, others have demonstrated lower PFJ forces during running (29), walking (45), and stair ascent (44), in adults with PFP when compared to asymptomatic cohorts. Within each of these studies, adults with PFP demonstrated a lower knee extension moment that drove the observed reduction in PFJ force. We did not observe a lower knee extension moment in the adolescents with PFP. A lower knee extension moment is likely the result of reduced quadriceps force, which has been proposed as a compensatory strategy to mitigate pain and symptoms associated with PFP (53). Previous research has suggested that age and symptom duration may affect motor adaptation (54). It is possible that adolescents may not possess the capacity to effectively modify their biomechanics in response to pain and thus did not demonstrate a difference in their PFJ forces when compared to asymptomatic adolescents (55).

Recommendations for PFP management advocate for strategies that lower PFJ forces (16, 17). Gait retraining interventions, such as an increase in cadence, are effective at lowering PFJ force and pain in adults with PFP (23, 47, 56). A 10% increase in cadence has been shown to lower impulse and, subsequently, lower cumulative and peak vertical GRF (57). A reduction in the magnitude of joint force with an increased step rate has been suggested to be effective at lowering microstructural joint damage often observed in lower limb injuries (25, 57). Although our cohort of adolescents with PFP ran with a higher cadence, there was no resultant change in peak PFJ force or cumulative PFJ load. Previous gait retraining studies that have adopted a higher cadence (i.e., ≥10%) have observed a reduction of 14%–19% in peak PFJ force (23, 47), and 12%–16% in PFJ stress (47, 58). In our cohort, the peak PFJ force was <5% different. This minor reduction did not compensate for the increased number of steps required to cover one km of running in our PFP group, resulting in no net benefit to cumulative PFJ force. These findings suggest that for cadence interventions to effectively lower cumulative PFJ force, they may need to achieve a higher reduction in peak PFJ force. Without this magnitude of change, the potential benefits of higher cadence may not be realised.

The cross-sectional nature of our study makes it unclear whether the higher running cadence in our PFP cohort was a response to pain. The average self-reported duration of symptoms by our cohort was approximately two years. This duration could have led to attempted motor adaptations such as the higher cadence to lower PFJ forces or mitigate pain. As discussed, the cadence may not have been high enough to have any beneficial biomechanical effect on PFJ forces but may be a pain avoidance strategy. Adults with longstanding PFP demonstrate greater hip adduction (19), greater contralateral pelvic drop (59, 60), and lower knee flexion angles (60, 61), during running, when compared to asymptomatic populations. This has not been observed in adolescents with PFP (22). It is possible that some biomechanical alterations in adults with PFP may develop in response to longstanding pain and/or as a response to changes in physical activity participation (62).

The finding of no differences in peak PFJ force between adolescents with and without PFP is consistent with studies of adults with PFP where no difference (51), or a lower peak PFJ force (29), has been observed during running in the symptomatic group. Despite not having elevated PFJ forces, treatment strategies aimed at reducing PFJ load have been shown to be effective at reducing pain in adults with PFP (56). Thus, it is possible a reduction in PFJ load may still provide a beneficial effect on pain and functional outcomes in adolescents with PFP and warrant further investigation. Symptom duration has also been shown to influence biomechanics (63), and prognosis (64, 65), in adults with PFP and future research may look to examine this in the adolescent cohort.

The findings of this study need be interpreted within the context of its limitations. Firstly, the model utilised to calculate PFJ force was based on a non-specific planar model constructed from previously published cadaveric data that involves the input of participant-specific parameters (i.e., knee joint flexion angle and knee extension moment) (46). This model does not consider individual patella geometry, contact area, patellar kinematics or the plantarflexor and hamstring muscle contribution. However, this model has been used in prior studies to estimate PFJ force (44, 45). Second, maturation stages can affect running biomechanics in asymptomatic adolescent cohorts (36, 66, 67). Although participant maturation stages were estimated, our sample size was not adequate to control for maturation as a covariate in this analysis. However, maturation stages were similar between groups and there were only three participants considered pre-pubertal. Future studies may look to examine adolescents across a range of maturation stages. Finally, we only examined differences between groups during a short bout of treadmill running. Studies have shown little differences in our variables of interest between overground and treadmill running (68, 69), but it is unclear if our results translate to longer durations of running, different velocities or across various running terrains.

In summary, adolescents with PFP do not demonstrate elevated PFJ forces during running when compared to asymptomatic adolescents. In addition, adolescents with PFP do not demonstrate changes in knee joint kinematics and kinetics during running when compared to adolescents who are pain-free. Prospective studies are required to evaluate the effect PFJ forces may have in the development of adolescent PFP.

## Data Availability

The original contributions presented in the study are included in the article, further inquiries can be directed to the corresponding author.
